# Prediction of Influence of Environmental Factors on the Toxicity of Pentachlorophenol on *E. coli*-Based Bioassays

**DOI:** 10.3390/s25103215

**Published:** 2025-05-20

**Authors:** Sulivan Jouanneau, Gerald Thouand

**Affiliations:** Nantes Université, Oniris, Centre National de la Recherche Scientifique, Génie des Procédés—Environnement—Agroalimentaire, Unité Mixte de Recherche 6144, F-85000 La Roche sur Yon, France; gerald.thouand@univ-nantes.fr

**Keywords:** bioassay, biomeasure, neural network, predictive approach, toxicity assessment

## Abstract

Evaluating the impact of pollutants on ecosystems and human health is crucial. To achieve this, a wide range of bioassays, using organisms of different trophic levels, are available. Extrapolating the results of these bioassays to real environmental conditions remains a major challenge. This study addresses this challenge by aiming to develop an algorithm capable of predicting the effect of environmental conditions on the impact of a toxicant, pentachlorophenol (PCP). Three abiotic factors were considered: pH, temperature, and conductivity. In the absence of the toxicant, the activity of *Escherichia coli* is influenced only by pH and temperature. However, exposed to PCP, the sensitivity of the bacteria was affected by these three factors. From these data, a predictive model was established to assess the intensity of the toxic effect induced by PCP. This model was validated using a validation dataset and demonstrated a strong correlation between the experimental and predicted values (r^2^ ≈ 0.9). Thus, this approach enables the effective prediction of PCP’s effects by accounting for environmental variations. This proof of concept constitutes a potential alternative, complementary to conventional models like BLMs (focused on water chemistry for metals) and QSARs (linking structure to intrinsic toxicity), which often overlook the complexities of real-world environmental conditions.

## 1. Introduction

Biomeasures such as bioassays and biosensors have become indispensable tools in the field of environmental monitoring. Indeed, these biological strategies can provide valuable information on environmental quality, particularly by enabling the characterization of the overall state of an environment (level of toxicity induced by anthropogenic pressure, toxicity) [1,2,3,4]. To achieve this, these approaches rely on bioindicators such as enzymes, cells (prokaryotic or eukaryotic), or certain multicellular higher organisms.

**Figure 1:**
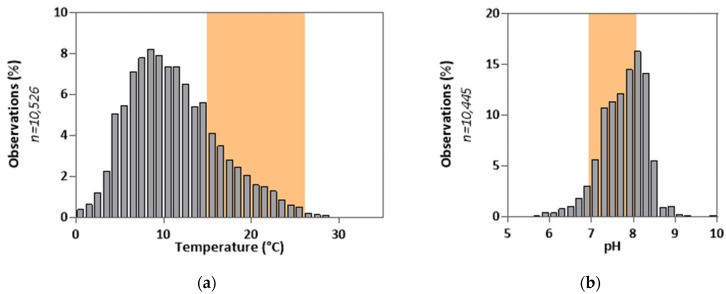
Physicochemical data collected in French surface water in 2019 [23]. The colored area corresponds to the covered range by the reference ecotoxicological methods [1]. (**a**) Temperature values collected in French surface water; (**b**) pH values collected in French surface water.

Despite their potential, current bioassay methods face several limitations. One of the main issues lies in the lack of representativeness of the data provided. Indeed, to ensure a certain level of reproducibility, these tests are carried out under controlled laboratory experimental conditions (pH, temperature, conductivity, etc.) (see Figure 1 and Table S1), implying, at the same time, a possible adjustment (correction) of the samples before their analysis (examples: prior salinity adjustment, temperature correction of samples) [5,6,7,8]. This technical choice in favor of reproducibility is in contradiction with the reality of the field. Indeed, adjusting the samples to the conditions imposed by the biological test can have significant consequences on the collected information, due in particular to the induced denaturation of the samples [9,10,11]. The work of Vasseur et al. [9], carried out on the basis of the ISO 11348 test (*Aliivibrio fischeri*) [12], highlights an increase in the toxic effects of cadmium (Cd^2+^) and zinc (Zn^2+^) following a 5 °C increase in the temperature of the tests (authors’ hypotheses: better adsorption of metals by cells and faster cellular metabolism). Conversely, the results show a decrease in the toxic effects induced by benzene under the same conditions (greater volatilization of the molecule under these conditions). In this context, the extrapolation of the results from these tests to an environmental reality can prove to be particularly complex, even hazardous, as the conditions are so different.

To attempt to address this issue, statistical models, such as Biotic Ligand Models (BLMs), have been developed to consider the specific characteristics of target environments in order to extrapolate observed effects to other specific environments [13,14,15,16,17]. To do this, the models require a thorough knowledge of the characteristics inherent to the environment studied (temperature, some aquatic ions (Ca^2+^, Mg^2+^, Na^+^, K^+^, Cl^−^, and SO_4_^2−^), sulfide, pH, alkalinity, and Dissolved Organic Carbon). In the current state of our knowledge, these models have been deployed exclusively to assess the effect of metals (no BLMs for organic compounds) but do not allow for the complexity of ecological interactions that can influence the toxicity of substances to be accounted for. Furthermore, although interesting, this strategy remains relatively expensive due to the necessary prerequisites (analytical data), making it poorly suited for regular environmental monitoring. Quantitative Structure-Activity Relationship (QSAR) models are also interesting predictive tools [18]. QSAR modeling allows for the prediction of the biological effects of chemical substances (such as toxicity) based on their molecular structure [19]. Although increasingly performant, these models require a significant effort in describing the molecular structure of the targeted molecules [20]. The main limitation of QSARs lies in their inherent difficulty in fully integrating the complexity and variability of real environmental conditions, limiting their deployment in an environmental monitoring context.

In the context of this study, we focused on the surface waters with a salinity of less than 35 g·L^−1^ (average salinity of seawater [21,22]). However, surface water analyses conducted by the French Office for Biodiversity [23] highlight significant physico-chemical disparities in these environments (Figure 1), notably induced by geographical factors (soil type, etc.), anthropogenic factors (industrial and agricultural activities), and/or seasonal variations. For example, temperature, pH, and conductivity values, observed during the 2019 survey across the national territory, range from 0 to 32 °C, 4.8 to 10.5, and 10 µS·cm^−1^ to 47 mS·cm^−1^ (conductivity close to seawater), respectively. The variability of these parameters must be taken into consideration to assess the effect of pollutant(s) in these environments. It is therefore necessary to propose solutions to transpose results obtained in the laboratory, under controlled conditions, to another environment subjected to physico-chemical conditions different from the test conditions.

The project proposed in this article aimed precisely to provide answers to this problem by using a supervised learning tool designed to predict the effect of a toxicant in a different environment subjected to specific physico-chemical conditions. To achieve this, we focused on a model organic pollutant, namely pentachlorophenol (PCP) (details in Supplementary Data). The challenge was to propose a model capable of predicting the toxic effect of this compound on the bacterial strain *Escherichia coli* based on three common abiotic factors: pH, temperature, and conductivity. Initially, the respiratory activity of the strain was characterized under varying pH, temperature, and conductivity conditions (three modalities per factor). Respiratory activity was monitored, according to the method proposed by Tizzard et al. in 2006 [24,25], using a redox marker: resazurin. This soluble and non-toxic indicator is reduced by electron transfer reactions associated with cellular respiration into a fluorescent byproduct (resofurin − λ_excitation_ = 571 nm, λ_emission_ = 585 nm) [26,27]. From the collected experimental data and using a “neural network” type approach (supervised learning approach), a predictive model was established.

## 2. Materials and Methods

### 2.1. Bacterial Strain

The study was conducted using the *Escherichia coli* K12 MG1655 strain (ATCC 700926). Cells are stored in cryotubes at −80 °C in a sterile suspension of glycerol (Sigma Aldrich, St. Louis, MO, USA, G9012) at 50% (*v*/*v*) in distilled water.

### 2.2. Growth Conditions

The medium used for cell growth was prepared as follows: 1 L of distilled water was supplemented with 5 g of NaCl (Carlo Erba, 479687, Val-de-Reuil, France), 5 g of yeast extract (Biokar Diagnotics, Allonne, France, A1202HA), and 10 g of tryptone (Biokar Diagnotics, A1404HA). Next, 15 g·L^−1^ of Agar (Biokar Diagnotics, A1012HA) was added in the liquid medium to obtain a solid medium used in the Petri dishes. The pH was adjusted to 7 with a solution of HCl (0.2 M–Sigma Aldrich, H1758) or NaOH (0.2 M–Sigma Aldrich, S8045), and the medium was sterilized by autoclaving at 120 °C for 20 min.

Cells were grown on Petri dishes containing solid medium at 30 °C for 12 h to obtain isolated colonies. Solid cultures were stored at 4 °C for a maximum period of 4 days.

Pre-cultures in liquid medium were prepared from isolated colonies on Petri dishes. After an overnight incubation (approximately 8 h) at 30 °C with shaking (250 rpm), a culture was performed in the same medium and under the same temperature and shaking conditions, but at an initial cell density of A_620 nm_ = 0.1. The density of the bacterial suspension was monitored until a density of A_620 nm_ = 0.45 was reached (absorbance value corresponding to the exponential growth phase of the bacterial strain).

### 2.3. Toxic Solutions

The pentachlorophenol (PCP) stock solution was prepared at a concentration of 10 g·L^−1^ from pentachlorophenol sodium salt (CAS number 131-52-2; Sigma Aldrich, 76480) in distilled water.

For the purposes of the study, different solutions (5 pH levels–5 conductivity levels) were prepared to be used as a matrix for the preparation of toxicant stock solutions. The pH of these different solutions was adjusted using a Sørenson’s phosphate buffer (67 mM) [28] composed of two solutions: a solution A of KH_2_PO_4_ at 67 mM (9.08 g·L^−1^; Sigma Aldrich, 795488) and a solution B of Na_2_HPO_4_ at 67 mM (9.47 g·L^−1^; Sigma Aldrich, 71640). These two solutions were prepared in distilled water. Table 1 summarizes the preparation conditions of the different matrices made for the study to achieve the target pH values.

The conductivity (at 25 °C) of the matrices was adjusted with powdered NaCl (Carlo Erba, 479687) to achieve the targeted levels (8.13, 11.03, 15.67, 18.14 and 22.17 mS·cm^−1^). Each condition was rigorously checked using a pH probe (PHC10101, Hach^®^, Loveland, CO, USA) and a conductivity probe (CDC40101, Hach^®^) coupled with a multimodal meter (HQ40d, Hach^®^).

PCP test solutions were prepared immediately before use, from the stock solution, in these different matrices at the following concentrations: 0, 10, 20, 50, 100, 200, 500, and 1000 mg·L^−1^.

### 2.4. Bioassays

Once the desired cell density was reached (A_620 nm_ = 0.45), the suspension was centrifuged for 5 min at 6400 RPM and 4 °C. The supernatant was removed, and the pellet was resuspended in sterile MgSO_4_ solution at 10^−2^ M maintained at 4 °C (2.46 g·L^−1^ of MgSO_4_∙7H_2_O—Sigma Aldrich, 63138). This washing step was performed twice. Finally, the washed pellet was resuspended in fresh liquid medium and the cell density was adjusted to A_620 nm_ = 1.

Bioassays were performed in white 96-well microplates (White plates, 96-MicroWell™, FluoroNunc™, Dutscher, Bernolsheim, France). In each well, 180 µL of test solution (PCP or control) were injected, to which 20 µL of the washed and adjusted bacterial suspension were added. The microplate was then incubated at the desired temperature (10 °C, 20 °C, or 30 °C) for 40 min. After this incubation step, 20 µL of resazurin (PrestoBlue™, Invitrogen™-Thermo Fisher Scientific, A13261, Waltham, MA, USA) were added to each well. Respiratory activity was monitored by fluorimetry using the SPARK^®^ multimode microplate reader (TECAN, Männedorf, Switzerland) (λ_excitation_ = 550 nm, bandwidth_excitation_ = 20 nm, λ_emission_ = 600 nm, bandwidth_emission_ = 20 nm, gain = 65, number of flashes = 30, integration time = 40 µS). Fluorescence measurements were performed every 10 min for 1 h under the same temperature conditions as the incubation.

### 2.5. Data Acquisition

**Figure 2:**
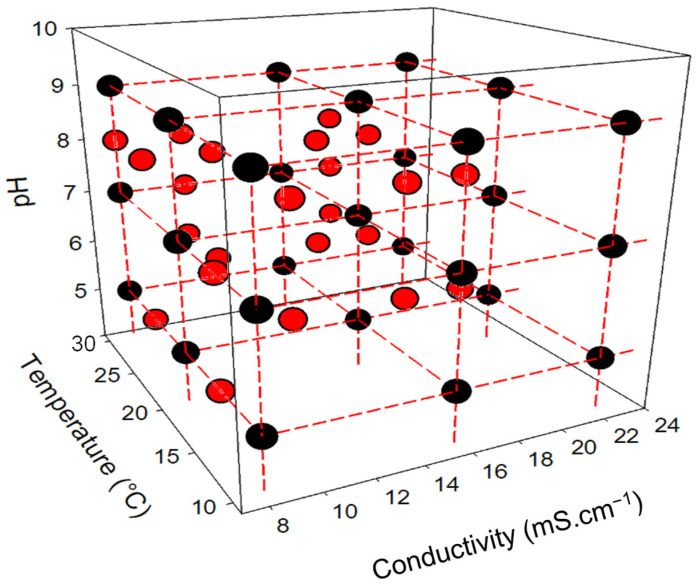
Experimental plan of the different tested conditions in the study framework (black circles: conditions used for the training dataset; red circles: conditions used for the validation dataset).

A first dataset was created specifically to train neural network models for data mining. This training dataset includes 216 experimental conditions, resulting from the combination of three factors (pH, conductivity, and temperature), representing 27 abiotic conditions (three levels per abiotic parameter) (see Figure 2), and eight levels of toxicant concentration. For each experimental condition, the raw data collected represent the respiratory activity of bacteria (monitored by fluorimetry) over time. Each condition was tested in three replicates to account for experimental variability, increasing the training dataset to 648 values.

A second dataset was created to validate the neural network models. To evaluate the robustness of these predictive models, 24 new combinations of abiotic factors were introduced (see Figure 2). Each condition was tested with the 8 PCP concentrations, in duplicate, resulting in a validation dataset of 384 values.

### 2.6. Data Processing

The entire process of data processing and model validation is schematically represented in Figures S1 and S2 in the Supplementary Data.

#### 2.6.1. Raw Data Pre-Processing

The raw fluorescence data, collected over time during the assays (see Section 2.4.), were modeled by linear regression (least squares method—Microsoft Excel) for each tested condition (a condition being defined by a specific pH value, conductivity, temperature, and PCP concentration). Thus, a respiratory activity value, corresponding to the slope of the calculated regression models, could be attributed to each tested condition. In total, 648 and 384 kinetic values were calculated for the training and validation datasets, respectively. These kinetic values, which represent relative fluorescence measurements over time, will therefore be expressed in arbitrary units (a.u.) for simplicity in the rest of this document.

These values were then converted into inhibition rates in order to determine the intensity of the toxic effect induced by each of the different tested conditions. To this end, the respiratory kinetics obtained for each condition were compared to a control (same combination of abiotic conditions but without PCP) in order to deduce an inhibition rate (percentage between 0% and 100%). The higher the inhibition value (closer to 100%), the stronger the intensity of the induced toxic effect. The equation used is described in Equation (1).

(1) $${INH\%}_{PCP-T{}^{\circ}C-pH-CS}=1-\frac{{\;Respiratory\;kinetic}_{PCP-T{}^{\circ}C-pH-CS}}{{\;Respiratory\;kinetic}_{control-T{}^{\circ}C-pH-CS}}$$

*INH%_PCP-T°C-pH-CS_*: inhibition rate calculated as a function of abiotic factor levels and PCP concentration.

*PCP*: concentration of PCP

*T°C*: temperature of assay (10, 20 or 30 °C)

*pH*: pH of sample (5, 7 or 9)

*CS*: conductivity of sample (8.13, 15.67 or 22.17 mS·cm^−1^)

#### 2.6.2. EC_50_ Determination

For each combination of abiotic conditions, EC_50_ values (half maximal effective concentration) were determined from the respiratory activities. Two non-linear regression models (Hill [29,30] and Weibull [31]) were used with the least squares method (MATLAB R2018b—MathWorks^®^, Santa Clara, CA, USA). Only the EC_50_ values from the model with the best correlation coefficient were retained for each combination of conditions.

#### 2.6.3. Development of Exploratory Neural Network Models

The neural network models were developed using the Neuro One 6.13.0.5 software (InModelia, Paris, France), chosen for its robust implementation of various neural network architectures and user-friendly interface for model development and evaluation.

The developed models were all based on the same architecture consisting of three layers of neurons. The first layer is in charge of data normalization so that each input variable has an equivalent weight. To achieve this, the different input data (pH, temperature, salinity, and PCP concentration values) are normalized by centering-reduction. This first neural layer is composed of as many normalization neurons as there are input data points (*n* = 4). The second layer, called the hidden layer, is the heart of the neural network. It allows the model to learn complex and hierarchical representations of the data by progressively transforming the normalized data through interconnected neurons. To ensure a certain robustness in the generated models and limit the risks of overfitting, the number of hidden neurons tested ranged from 1 to 3. For each architecture tested, 10 models were generated using three different activation functions (hyperbolic tangent, arctangent, and exponential). The last layer is the output layer; it ensures the interpretation of the data provided by the previous layer (hidden layer) in order to propose a numerical value (respiratory activity or inhibition rate). To achieve this objective, this neuron also integrates an activation function, but unlike the hidden neurons, this activation function is linear. In other words, the value provided by this neuron corresponds to the weighted sum (weighting by iteration) of the information coming from the hidden neurons.

During the training phase, 10 models were generated based on the different imposed architectures (1–3 hidden neurons; 3 types of activation function). These models were generated by iteration (100 iterative cycles per model) and selected based on their representativeness according to two criteria: the standard deviation and the coefficient of determination (r^2^) calculated between the modeled data and the real data.

To evaluate the effect of abiotic factors on bacterial respiratory activity, an initial set of models (90 models) was established based on the respiratory kinetics of the training dataset, but excluding all conditions with a non-zero toxicant concentration (i.e., 81 values) (Figure S1). The generated models were then compared based on their ability to best represent the experimental data.

For the second part of the study aimed at establishing a link between abiotic conditions and the intensity of the toxic effect induced by PCP on *E. coli*, we performed a second modeling step based, this time, on the entirety of the training dataset (*n* = 648). For this second phase, we used, as output data, the calculated inhibition rates (indicator of the intensity of the induced toxic effect). The most relevant effect model, among the 90 new models established, was selected based on the coefficient of determination calculated from the training data. To validate the selected effect model, we used the validation dataset (cf. 2.5) based exclusively on novel abiotic combinations (384 inhibition rates). For this, the conditions tested in the validation dataset were submitted, in parallel, to the predictive models, thus generating a modeled dataset. The data from the validation dataset were compared to those of the modeled dataset to calculate the coefficient of determination and deduce the predictive capabilities of the neural model (Figure S2).

#### 2.6.4. Complementary Statistical Analyses

Complementary statistical analyses (Spearman’s non-parametric correlation test and calculation of the coefficient of determination r^2^) were performed using Prism 6 software (version 6.07, GraphPad, San Diego, CA, USA).

## 3. Results

### 3.1. Effect of Abiotic Parameters (Temperature, pH, Conductivity) on the Respiratory Activity of the E. coli Strain

**Figure 3:**
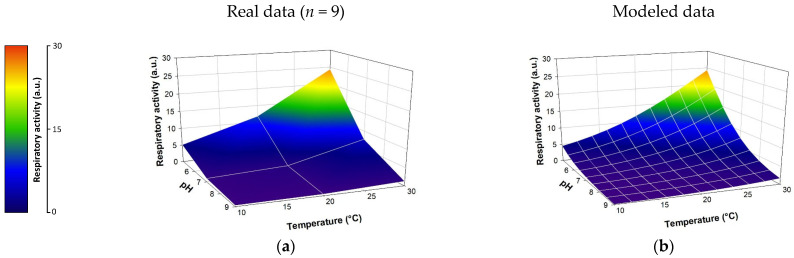
Modelling of the respiratory activity of *E. coli* according to the abiotic factors (temperature and pH). The model expression is detailed in Supplementary Data (see Equation (S1)). (**a**) Mean of experimental data (mean variability between replicates = 31%); (**b**) modeled data. a.u.: arbitrary units.

As expected, certain abiotic parameters play a major role in the biological response. Indeed, under ‘favorable’ conditions of temperature (30 °C), pH (5), and conductivity (8 mS·cm^−1^) and in the absence of toxicant, microorganisms show maximum respiratory activity (see Figure 3; respiratory activity = 25.05 a.u.). Statistical analyses performed show also that two of the three abiotic factors tested have a significant effect on biological activity (α = 0.05), namely, temperature (Spearman’s r = 0.45, *p*-value < 0.0001) and pH (Spearman’s r = −0.83, *p*-value < 0.0001). For example, an increase in pH from 5 to 9 can induce a decrease in the respiratory activity of the *E. coli* strain by a factor of 20.2 ± 4.5. Similarly, a decrease in the assay temperature from 30 °C to 10 °C divides the measured respiratory intensity by 6 ± 1.9 (Figure 3). According to the data, conductivity does not significantly affect (Spearman’s r = −0.13, *p*-value = 0.24) the respiratory activity of the *E. coli* strain in the tested range (8.13 mS·cm^−1^ to 22.17 mS·cm^−1^). Given that there is no statistically significant impact of conductivity on the respiratory activity of the bacteria, all conditions presenting identical temperature and pH values were averaged to establish Figure 3. This grouping of conditions artificially increased the number of replicates from three to nine, also increasing the average observed variability from 3.09% to 31%.

The intensity of *E. coli* respiratory activity as a function of significant abiotic parameters was modeled using a neural network approach via Neuro One 6.13.0.5 software (InModelia). Several architectures and neural activation modes were tested. The selected model relies on a hidden layer consisting of a single neuron, activated by a ‘hyperbolic tangent’ function (Equation (S1)). Figure 3 shows the respiratory kinetic raw data as well as the modeled data. The coefficient of determination r^2^ between the average experimental values and the modeled values is 0.944. According to these results, the neural model represents the action of abiotic parameters on biological activity with high fidelity. It is thus possible to predict the effect of abiotic modifications on the respiratory activity of an *E. coli* bacterial strain.

### 3.2. Modeling of Toxic Effects Induced by PCP as a Function of Abiotic Parameter Levels

#### 3.2.1. Combined Effects of Abiotic Parameters (Temperature, pH, Conductivity) on the Inhibition of *E. coli* Respiratory Activity Induced by PCP

Eight PCP concentrations were tested to evaluate the effect of abiotic conditions on the bacterial strain’s sensitivity. As expected, the presence of the toxicant induces a significant inhibition of bacterial respiratory activity. The EC_50_ obtained for PCP at 30 °C, pH = 5, and 8.13 mS·cm^−1^ was estimated at 7.3 mg·L^−1^ (±0.01 mg·L^−1^). However, this indicator varies depending on the abiotic conditions. At pH = 7 and pH = 9, the EC_50_ value significantly increases to 42.01 mg·L^−1^ (±1.9 mg·L^−1^) and 190.2 mg·L^−1^ (±5.98 mg·L^−1^), respectively, which can be interpreted as a decrease in the sensitivity of the *E. coli* strain to PCP (membrane permeability, metabolic activity, etc.) and/or a decrease in the toxic effect induced by PCP (bioavailability, chemical form, etc.).

Spearman’s correlation analysis shows a significant correlation (α = 0.05) between the calculated inhibition rates and the different abiotic factors tested. As expected, the ‘concentration’ factor is very strongly correlated with the inhibition rates (r_(concentration)_ = 0.71, *p*-value < 0.0001). This strong correlation is explained by the expected dose-response effect. The ‘pH’ factor is inversely correlated with the biological response (r_(pH)_ = −0.43, *p*-value < 0.0001). In other words, the higher the pH value (within the tested range), the lower the observed inhibition rate. This observation is consistent with the work of Rutgers et al. [32], which showed similar effects of pH on the action of PCP (growth monitoring of *Sphingomonas* sp.). PCP is a weak acid (pKa_PCP-sodium salt_ = 5.3). In water, two forms coexist depending on the pH, PCP^0^ (C_6_Cl_5_OH, liposoluble, and predominant when pH < 5.3) and PCP^−^ (C_6_Cl_5_O^−^, the dissociated anionic form, highly soluble in water, phenolate ion, predominant when pH > 5.3) [33]. PCP acts as an uncoupler of oxidative phosphorylation. This process, occurring at the level of the inner membrane of bacteria, induces the inhibition of ATP production, explaining the toxic effect observed on *E. coli* [34]. Consequently, it is necessary for the molecule to enter the intracellular environment to exert its toxic effect. In its PCP^0^ form, PCP is more lipophilic and therefore more easily crosses the lipid membrane of bacteria to penetrate the cell. Conversely, the PCP^−^ form crosses the membrane with greater difficulty (electrostatic repulsions, lower lipophilicity), thereby limiting its toxic impact. The form taken by PCP in water can explain the observed decrease in toxicity when the pH increases. Beyond the phenomenon of dissociation, pH can also play a significant role in the functioning of the cell, particularly on plasma membranes [35]. Thus, by modifying the permeability characteristics of the membrane, it can impact the bioavailability of PCP for the cells and therefore modify the intensity of the induced toxic effect.

To a lesser extent, the ‘conductivity’ and ‘temperature’ factors also contribute to the biological response (r_(conductivity)_ = 0.20, *p*-value ≤ 0.0001; r_(temperature)_ = 0.16, *p*-value ≤ 0.0001). Conductivity (an indicator of salt concentration) plays a predominant role in maintaining the cell’s osmotic balance. The increase in conductivity of the extracellular environment can induce potential osmotic stress for the cell. In response to this stress, the bacterial strain will implement ATP-dependent biological mechanisms [36]. Thus, osmotic stress could increase the ATP requirements, already compromised by the action of PCP. An increase in conductivity tends also to modify the lipid structure [37] and increase the permeability [38] of bacterial membranes. This phenomenon can facilitate the diffusion of the toxicant into the intracellular environment and, consequently, increase the induced toxic effect, thus explaining the results obtained. In parallel, temperature increases the fluidity of cellular membranes [39,40], which can increase their permeability to toxicants. By facilitating the entry of toxicants into bacterial cells, the intensity of the induced effect can be modified [41].

#### 3.2.2. Development and Validation of Predictive Models

The training dataset (*n* = 648) was used for the development of predictive models (neural network models) aiming to correlate the inhibition rate of the *E. coli* strain as a function of PCP concentration and abiotic factors (pH, conductivity and temperature). Several neural architectures were tested based on a variable number of hidden neurons and with different neural activation modes.

**Figure 4:**
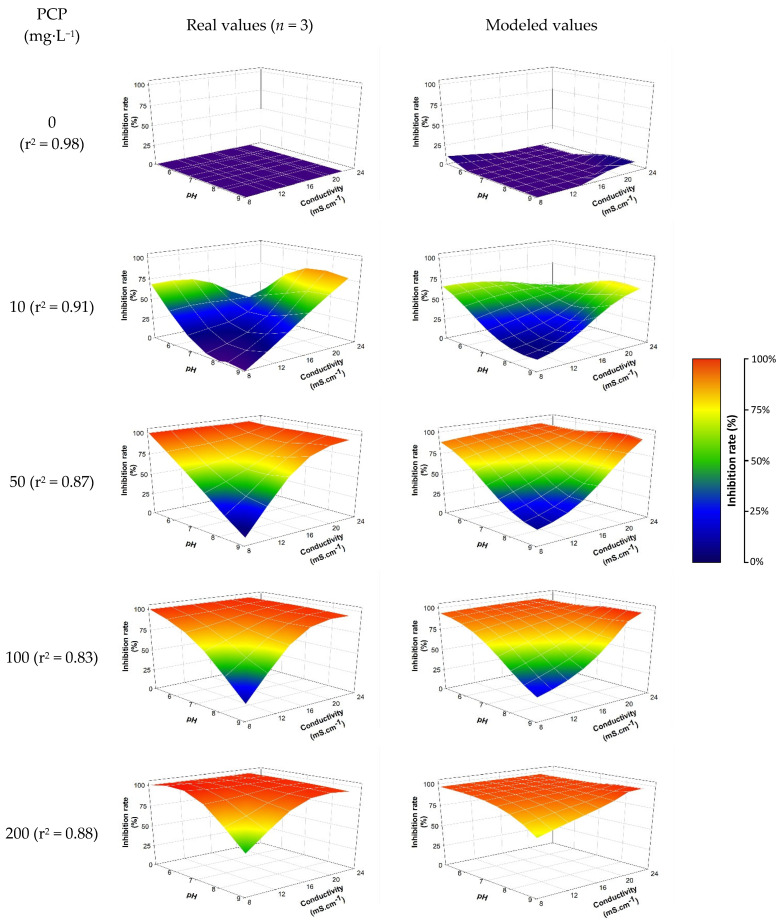
Average observed/modelled inhibition rate according to pH and conductivity at 30 °C and the PCP concentration (mean variability between replicates = 4.1%). The model expression is detailed in the Supplementary Data (see Equation (S2)).

The neural model selected from the 90 generated models relies on three hidden neurons activated by ‘hyperbolic tangent’ functions (Equation (S2)). The intensity of toxic effects (inhibition rate) as a function of abiotic parameters is shown in Figure 4. The coefficient of determination (r^2^) between the experimental data and the modeled data from the selected neural algorithm is estimated at 0.918. The selected modeling allows for the establishment, with a certain relevance, of the relationship between the biological response (intensity of the toxic effect) and the levels taken by the studied abiotic parameters (pH, conductivity, temperature). The results notably allow for the visualization of the effect of abiotic parameters as mentioned previously. As expected, pH plays a predominant role in the biological response (see Section 3.2.1): the higher the pH, the less significant the toxic intensity induced by PCP. However, the combined effect of a decrease in pH associated with an increase in conductivity reinforces the toxic effect generated by PCP. For a PCP concentration of 50 mg·L^−1^, the observed inhibition is approximately 9.44% ± 1.2% versus 99.56% ± 0.07% for pH = 9 and conductivity = 8.13 mS·cm^−1^ and pH = 5 and conductivity = 22.17 mS·cm^−1^, respectively. Temperature also impacts the induced toxic intensity but to a lesser extent.

**Figure 5:**
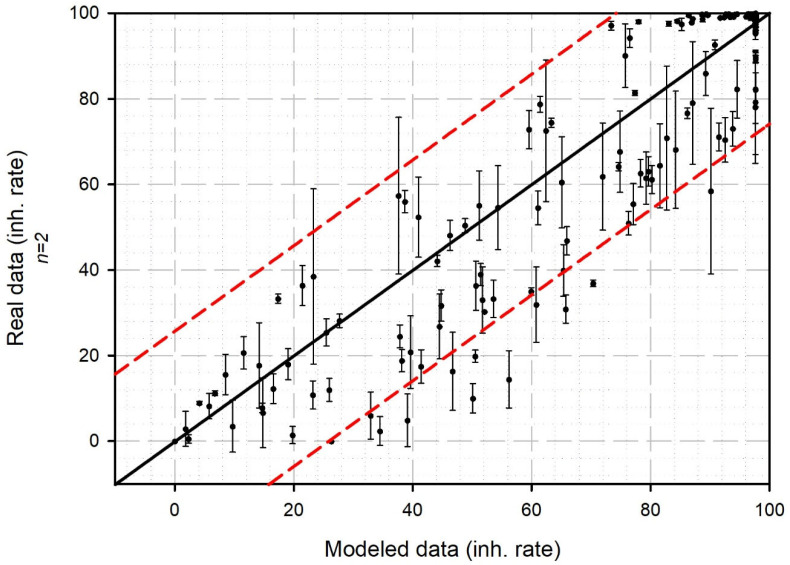
Validation of the predictive model performed using the validation dataset (*n* = 384). Black line: correlation line; red dashed line: 95% prediction band.

To validate the approach, the selected model was deployed on novel conditions included in the validation dataset (abiotic parameter levels different from those used in the training dataset). The results thus generated were compared to the collected experimental data (Figure 5). An r^2^ of 0.8877 suggests that the predicted results are consistent with reality. Indeed, the model is able to predict, with a certain robustness, the intensity of toxic effects likely to be observed experimentally.

Consequently, these results validate the idea of extrapolating results from a bioassay. Indeed, these results highlight the ability, through a predictive model, to predict the effect of a pollutant on a target organism, taking into account significant environmental variations. Therefore, it is feasible to transpose results obtained in the laboratory under controlled conditions to another environment by weighting the response according to the levels taken by the abiotic factors of the target environment.

This demonstration, however, remains limited in terms of deployment. Indeed, the chosen predictive model, being based on a supervised learning approach (iterative neural network approach), the scope of application remains confined to the tested pollutant, i.e., PCP, and the bacterial strain used (*E. coli*). Furthermore, abiotic effects play a combined role on cellular metabolism but also on the physico-chemistry of pollutants [42,43]. Also, in the current state of knowledge, extrapolation to other molecules remains very complex and requires a better understanding of the environmental chemistry of pollutants [44]. Finally, the resistance mechanisms likely to be deployed by exposed organisms are very diverse, inducing very different resistance capacities from one organism to another [45,46].

## 4. Discussion and Conclusions

Through this study, a model was established by supervised learning (neural network) allowing to predict the level of toxicity induced by an organic molecule (PCP) on an organism (*E. coli*) with relevance (r^2^ ≈ 0.9). The major interest of this model lies in its ability to take into account environmental variations related to pH, temperature, and/or conductivity. Although limited to a single model organic pollutant, this study constitutes a proof of concept demonstrating that it is possible to anticipate the effect of a toxicant, PCP, in different environments by weighting its action according to simple abiotic parameters.

The developed model possesses inherent limitations regarding its direct applicability to other molecules. This constraint stems from the intricate interplay between abiotic factors and the toxicant-target relationship. As highlighted earlier, environmental parameters such as pH, temperature, and conductivity exert a significant influence on both the biological sensitivity of the target organism (in this case, *E. coli*) and the chemical behavior of the toxicant itself. The interactions between abiotic factors, target organisms, and pollutants necessitate the use of data-driven methodologies. Supervised learning approaches, such as the neural network model employed in this study, offer a powerful means to capture these complex relationships by learning from empirical data. However, the model’s reliance on training data specific to PCP and *E. coli* limits its immediate transferability to other molecules or organisms. Indeed, the toxicity of PCP is very significantly affected by environmental abiotic conditions, particularly pH. The empirical model in this study was developed exclusively based on the toxic effects induced in bacteria. Consequently, the model does not integrate this theoretical dimension related to the form of PCP induced notably by pH. Also, to consider transposing it to other molecules, significant work will need to be undertaken to implement these models with characterization data such as pKa, solubility, or Kow. Regarding the transition of the model to other organisms, the challenge is quite different. Indeed, the biological response is directly linked to the inherent resistance capacities of each species and inscribed in its genome. Predicting the effect of a pollutant from one species to another is a very complex exercise without going through an experimental phase [47,48]. Therefore, work will also need to be carried out to attempt to integrate this complex biological dimension into the proposed models.

This proof of concept, focused on a specific model pollutant and organism, presents a potential avenue for improving environmental pollutant management by suggesting new strategies likely to contribute significantly to its improvement. The proposed predictive tools open up new perspectives by enabling the extrapolation of laboratory findings to specific environmental contexts. Currently, regulatory decisions regarding pollutant management often rely on standardized bioassays conducted under controlled laboratory conditions. However, these conditions may not accurately reflect the environmental complexity. Abiotic factors, such as pH, temperature, and conductivity, can significantly influence the toxicity of pollutants, rendering laboratory-derived toxicity values potentially misleading. Consequently, legislators may be inclined to propose regulations intended to protect the environment based on laboratory findings, without considering environmental specificities. In the case of this study, the results obtained highlight the significant influence of pH and conductivity on PCP toxicity. Thus, these results directly suggest that regulatory bodies could implement targeted restrictions on the application or discharge of PCP in vulnerable environments such as acidic or high-conductivity waters. This capacity to adapt regulations according to specific environmental characteristics could provide a potentially more effective and environmentally protective approach than relying solely on toxicity values derived from controlled laboratory conditions. In the future, these tools could potentially be made available to industrialists, which might enable them to develop a more comprehensive understanding of the environmental effects of used molecules. They could then consider using these predictive tools to proactively assess and manage the environmental risks associated with their discharges in a more context-specific and responsible manner.

By integrating environmental variability into toxicity predictions, these tools may contribute to more informed decision-making regarding pollutant regulation and industrial practices.

## Figures and Tables

**Table 1:** pH of matrices used to prepare dilute toxic solutions.

	pH = 5	pH = 6	pH = 7	pH = 8	pH = 9
Solution A	98.8%	86.8%	38.5%	6%	0.3%
Solution B	1.2%	13.2%	61.5%	88%	99.7%

## Data Availability

The data are available on request to corresponding authors.

## Supplementary Materials

The following supporting information can be downloaded at: https://www.mdpi.com/article/10.3390/s25103215/s1, Figure S1: Procedure followed for raw data processing and neural network model development based on the learning dataset; Figure S2: Validation strategy for the predictive model developed from the training dataset; Table S1: Non-exhaustive list of test conditions for standardized tests dedicated to the evaluation of freshwater toxicity. References [49,50,51,52] are cited in Supplementary Materials.
