# Supplementary material for: Prediction of Influence of Environmental Factors on the Toxicity of Pentachlorophenol on E. coli-Based Bioassays
Source: Sensors (Basel). 2025 May 20;25(10):3215. doi: 10.3390/s25103215 (PMC12115939; doi:10.3390/s25103215)
Supplement: Supplementary file 1 [file sensors-25-03215-s001.zip › sensors-3548916-supplementary.pdf]

1. Supplementary Data

Table S1. Non-exhaustive list of test conditions for standardized tests dedicated to the evaluation of freshwater toxicity.

Target organism	Trophic level	Measurement	Standard	Laboratory conditions		
				Temperature (°C)	pH	Salinity (g/L)
Aliivibrio fischeri	Bacteria	Bioluminescence inhibition	ISO 11348	15	7 ± 0.2	30
**Activated sludge**	Wild consortium	Respiratory inhibition	ISO 8192	20 ± 2	7.5 ± 0.5	-
Pseudomons putida	Bacteria	Growth inhibition	ISO 10712	23 ± 1	-	-
Brachionus subspicatus	Rotifer	Growth inhibition	ISO 20666	25 ± 1	7.6 ± 0.3	-
Desmodesmus costatum	Freshwater microalgae	Growth inhibition	ISO 8692	23 ± 2	7.5	-
Skeletonema sp. and *Phaeodactylum tricornutum*	Marine algae	Growth inhibition	ISO 10253	20 ± 2	6.5	22
Daphnia magna Straus	Microcrustacean	Mobility inhibition	ISO 6341	20 ± 2	-	-
Ceriodaphnia dubia	Microcrustacean	Reproduction inhibition	ISO 20665	25 ± 1	8 ± 3	-[1]
Brachydanio rerio	Fish	Mortality	ISO 7346	23 ± 1	-	-

Environment		
[0; 32]	[4.8; 10.5]	[0.013; 34.25]

[1]: *Not applicable to the testing of aquatic samples from the estuarine or marine environment.*

[2]: *Estimated value in g/L based on conductivity data according to the method described in the book of Aminot and Kérouel* [49].

PCP details

Since the 1930s, PCP has been widely used for its fungicidal and bactericidal properties (notably as a wood preservative, fungicide, algaecide, etc.) [50]. It is classified as a Group 2B possible human carcinogen by the IARC and as a probable human carcinogen by the U.S. EPA [51]. Furthermore, several studies show that PCP is also an endocrine disruptor likely to interact with thyroid hormones and have effects on fetal growth and development in humans. Also, the use and production of PCP has been restricted or banned in many countries worldwide because of its adverse effects on the ecological environment and humans [52]. In France, its use has been prohibited since 2009, despite which significant concentrations have been found in different environmental compartments (> 200 µg.kg^{-1} in sediments, > 50 µg.L^{-1} in water) [23].

Eq. S1. Equation of the established predictive model Ra = f(T, pH).

$$\mathrm{Ra} = 24.85 - 24.96 \times \tanh(1.398 + 0.6589 \times (0.6184 * \mathrm{pH} - 4.345) - 0.4533 \times (0.1237 \times \mathrm{T} - 2.49))$$

Ra*: Respiratory activity (u.a.).*
pH*: Hydrogen potential of the targeted environment.*
T*: Temperature of the targeted environment (°C).*

Eq. S2. Equation of the established predictive model IR = f(C, pH, [PCP]).

$$IR = ((0.2746 - 0.566 * \tanh(0.3345 * (0.1746 * \mathrm{CS} - 2.706) - 27.32 - 0.2899 * (0.6184 * \mathrm{pH} - 4.345) + 0.1007 * (0.1237 * \mathrm{T} - 2.49) - 38.31 * (0.003048 * [\mathrm{PCP}] - 0.7162)) - 0.3098 * \tanh(3.902 - 1.061 * (0.1746 * \mathrm{CS} - 2.706) - 1.382 * (0.6184 * \mathrm{pH} - 4.345) - 0.2216 * (0.1237 * \mathrm{T} - 2.49) + 2.982 * (0.003048 * [\mathrm{PCP}] - 0.7162)) + 0.4406 * \tanh(2.185 + 0.3805 * (0.1746 * \mathrm{CS} - 2.706) - 0.914 * (0.6184 * \mathrm{pH} - 4.345) + 0.07101 * (0.1237 * \mathrm{T} - 2.49) + 3.258 * (0.003048 * [\mathrm{PCP}] - 0.7162))) - 0.1334)/0.8581$$

IR*: Inhibition rate (%).*
CS*: Conductivity of the targeted environment (mS.cm^{-1}).*
pH*: Hydrogen potential of the targeted environment.*
T*: Temperature (C).*
$[\mathrm{PCP}]$*: Concentration of PCP (mg.L^{-1})*

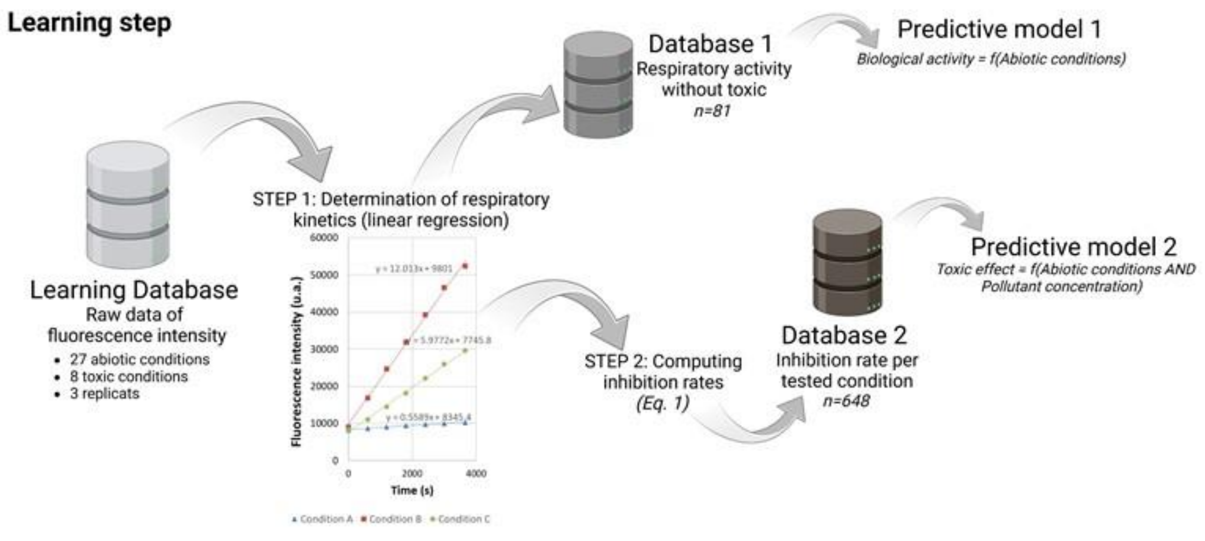

Figure S1. Procedure followed for raw data processing and neural network model development based on the learning dataset.

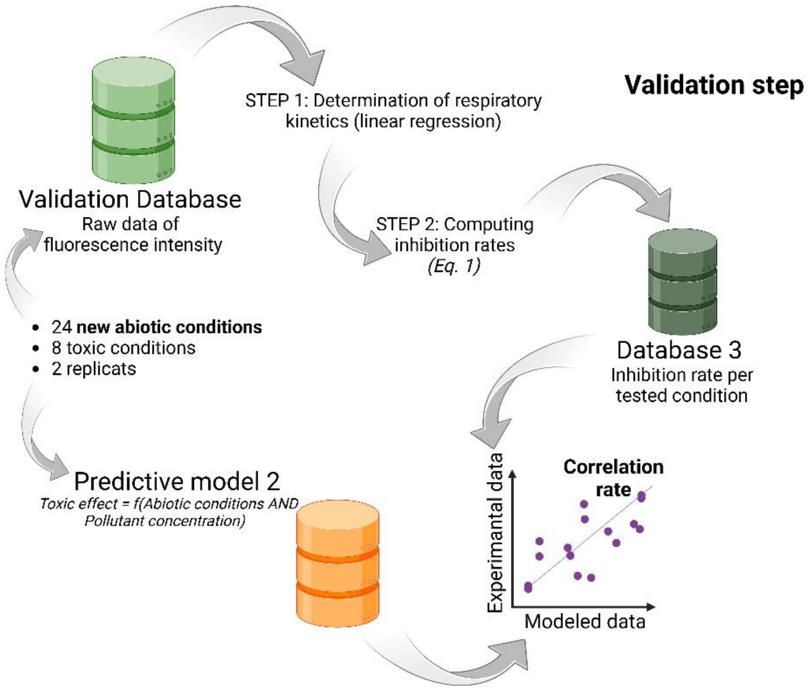

Figure S2. Validation strategy for the predictive model developed from the training dataset.

2. Bibliography

23. Office Francais de la Biodiversité Bienvenue sur Naïades|Naïades. Available online: https://naiades.eaufrance.fr (accessed on 24 July 2020).
49. Aminot, A.; Kérouel, R. *Hydrologie des Écosystèmes Marins: Paramètres et Analyses*; Editions Quae: Versailles, France, 2004; ISBN 978-2-84433-133-5.
50. Zheng, W.; Wang, X.; Yu, H.; Tao, X.; Zhou, Y.; Qu, W. Global Trends and Diversity in Pentachlorophenol Levels in the Environment and in Humans: A Meta-Analysis. *Environ. Sci. Technol.* **2011**, *45*, 4668–4675. https://doi.org/10.1021/es1043563.
51. U.S. California Environmental Protection Agency. *Public Health Goal for Pentachlorophenol in Drinking Water*; Office of Environmental Health Hazard Assessment: Sacramento, CA, USA, 1997.
52. European Union. *Commission Regulation (EC) No 552/2009 of 22 June 2009 Amending Regulation (EC) No 1907/2006 of the European Parliament and of the Council on the Registration, Evaluation, Authorisation and Restriction of Chemicals (REACH) as Regards Annex XVII (Text with EEA Relevance)*; European Union: Brussels, Belgium, 2009; Volume 164.
